# The Direct and Indirect Effects of Online Social Support, Neuroticism, and Web Content Internalization on the Drive for Thinness among Women Visiting Health-Oriented Websites

**DOI:** 10.3390/ijerph17072416

**Published:** 2020-04-02

**Authors:** Nikol Kvardova, Hana Machackova, David Smahel

**Affiliations:** Faculty of Social Studies, Masaryk University, 60200 Brno, Czech Republic; hmachack@fss.muni.cz (H.M.); smahel@fss.muni.cz (D.S.)

**Keywords:** drive for thinness, health-oriented websites, online social support, neuroticism, web content internalization

## Abstract

One of the debates about media usage is the potential harmful effect that it has on body image and related eating disturbances because of its representations of the “ideal body”. This study focuses on the drive for thinness among the visitors of various health-oriented websites and online platforms because neither has yet been sufficiently studied in this context. Specifically, this study aims to bring more insight to the risk factors which can increase the drive for thinness in the users of these websites. We tested the presumption that web content internalization is a key factor in this process, and we considered the effects of selected individual factors, specifically the perceived online social support and neuroticism. We utilized survey data from 445 Czech women (aged 18–29, M = 23.5, SD = 3.1) who visited nutrition, weight loss, and exercise websites. The results showed a positive indirect link between both perceived online social support and neuroticism to the drive for thinness via web content internalization. The results are discussed with regard to the dual role of online support as both risk and protective factor. Moreover, we consider the practical implications for eating behavior and weight-related problems with regard to prevention and intervention.

## 1. Introduction

Considering Western culture and its orientation toward appearance, young girls and women are susceptible to the desire to be thin so they would achieve an ideal body shape [1,2]. According to the Tripartite Influence Model [3], women internalize idealized thin body shapes from the media, which includes traditional mass media and the internet, including health-oriented websites. Exposure to thin-ideal content can have a negative impact on women because it is associated with their drive for thinness and eating disturbances [4,5].

In this study, we focus on the drive for thinness, which is motivation for a thin or thinner body and the desire to lose weight [1,6]. It is considered a risk factor for well-being because it is associated with decreased psychological health and the later development of anorexia and bulimia nervosa [7,8]. Because of its potential harm, it is crucial to understand the factors that are associated with the drive for thinness. Although previous studies investigated the role of the media in relation to the drive for thinness [1,5,9], there is a lack of evidence for health-oriented websites and the role they play in promoting weight loss. We intend to contribute to this area by focusing on these types of websites within the theoretical framework of the Tripartite Influence Model [3]. Moreover, our aim is to enrich this model, which posits socio-cultural influences on eating disturbances, by including the role of the individual factors associated with the drive for thinness. Specifically, we examine the role of these websites for the perceived online social support, neuroticism, and internalization, and their direct and indirect effects on the drive for thinness.

As a result, our aim is to extend the knowledge about the role of health-related websites in the development of eating disorders by showing how and for whom these online spaces pose a risk. Based on our conclusions, we propose recommendations for prevention and intervention efforts.

### 1.1. Drive for Thinness and Health-Oriented Websites

The drive for thinness is a motivational orientation toward having a thin or thinner body and a desire to lose weight [1,6]. It emerges as a motivated behavior in order to reduce body-related discontent [10], which is manifested by eating restraint and a preoccupation with body shape and weight [11]. It is considered a risk factor to women’s health because it is associated with decreased psychological well-being, like body dissatisfaction [10], body-related anxiety [12], lower self-esteem [8], or perceived stress [13]. Moreover, the drive for thinness is one of the diagnostic criteria for anorexia and bulimia nervosa, and it is associated with the later development of both [7,8,11]. The ideal of thinness [1], the drive for thinness, and related eating disorders are more prevalent in women than in men [14]. Therefore, we focused the study on women.

Considering the potential detrimental effects, it is important to understand the factors which exacerbate the drive for thinness. According to the Tripartite Influence Model [3], there are three main influences on disordered eating: parents, peers, and the media. The role of the media has been highly debated in relation to disordered eating. In the past two decades, substantial attention has been given to the role of new technologies, such as social networking sites, eating- and exercise-related websites, personal blogs with pro-eating disorder content, and various health-related discussion forums [15]. We focus on websites related to weight loss, nutrition, and exercise. These websites act as important sources for general online information related to nutrition, fitness, weight loss, and a healthy lifestyle. There are plenty of websites that address these topics, including personal blogs, informational websites for particular health-related themes, discussion forums, and social-networking groups [16,17]. Websites can be focused on weight loss, body shaping, healthy lifestyle, eating, dieting, nutrition plans for specific illnesses, recipes, and exercising [15,18,19]. Visitors may go through content, read articles, make and read comments, and obtain advice and inspiration. Moreover, websites can serve as a social environment where people interact with messages, comments, and evaluations, and they are places where people can receive support from other visitors [20,21,22].

However, these websites can have a negative impact on women because they display content that is associated with the drive for thinness, body dissatisfaction, and eating disturbances [4,5,23,24]. Specifically, some of these websites display pro-ED (pro-eating disorder) content that suggests that maintaining an eating disorder is a positive lifestyle choice [25]. They also contain positive comments about being thin, guilt-inducing messages related to food, stigmatization about weight, and expressions of negativity about being fat or overweight. They include content related to dieting and eating restraint, and the promotion of a thin-ideal appearance [18,26]. This appearance-oriented content can have a negative effect on women through the maintenance of weight- and appearance-related concerns [27].

The current study focuses on young female visitors of health-oriented websites in the Czech Republic. According to the data from Eurostat [28], 54% of Czech women aged 16 to 29 searched for online health-related information in 2016, which is the year when the data for our study was collected. The European average during that time was 60% of young women. Concerning the general usage of the internet, 95% of Czech women aged 16 to 29 stated that they used the internet in the preceding three months in 2016, whereas the European average was 96% [29]. This means that the usage of the internet and the online health seeking behavior among Czech women is similar as in other European countries.

### 1.2. Internalization

The negative effect of the exposure to the appearance-related online content can be explained with the Tripartite Influence Model, which suggests that the link between exposure to media ideals and eating disorders is not direct. It proposes that internalization of the appearance ideals serves as a mediating factor interfering association between media effect and disordered eating [30]. Media impact on disordered eating via internalization, as proposed by Tripartite Influence Model, was examined and supported by previous studies [31,32,33,34,35]. In the context of developing and maintaining eating disturbances, internalization is the process of adopting socially and culturally defined norms about body shape, which are commonly maintained as body ideals in everyday social interactions and in the media. By internalizing these ideals, one’s conception of self could be affected because the ideals can come to represent personal standards against which one could appraise self and others [34]. Since the idealized appearance depicted by the media does not always correspond with one’s real body shape, inconsistencies can emerge between the internalized norm and the actual body. Internalized ideals and perceived discrepancies can lead to consideration about how to obtain this ideal body [1]. This in turn results in disordered eating.

Several studies investigated specifically drive for thinness and how it is related to internalized appearance ideals in adolescent girls and young adult women. Internalization is a significant factor associated with the drive for thinness in both categories [1,8,15,35,36]. Moreover, the mediational role of internalization in the association between media exposure and the drive for thinness was supported [15,35]. However, less attention has been given to the individual factors which may be salient in this process and help explain who is susceptible to internalize media content. Therefore, in this study, we focus on two factors: online social support and neuroticism.

### 1.3. Online Social Support

Research has shown that seeking support from others is a frequent motivation for using health-oriented websites and participating in health-related online groups [37,38,39]. The online space offers various ways to get in touch with others, so there are also diverse ways to seek help and receive support. Social support, which in this context is mostly provided as emotional support, is expressed through emotions, empathy, and as informational support, like sharing knowledge regarding eating or fitness activities [21,40].

Online social support has been investigated as an important factor among people who struggle with eating disorders. For instance, women who engaged in an internet weight loss community mentioned encouragement, motivation, information, and shared experiences as significant resources. They appreciated the accessibility, the anonymity, and the non-judgmental interactions as unique characteristics of internet-mediated support [21]. Moreover, examinations of ED discussion forums and ED-oriented support groups have revealed that these online sites provide relevant information, emotional support, personal disclosure, help, friendship, peer support, and a safe space to ventilate feelings [20,22,39,41].

Though receiving social support is, in many occasions, a very beneficial process, we also examine its potential for the reinforcement of the drive for thinness via increased internalization. This process can be described with two theories: Social Identity Theory, which refers to an individual’s knowledge of belonging and the perceived emotional and value significance of group membership [42]; and the Self-Categorization Theory [43,44], which depicts how membership in social groups affects an individual’s behavior. Social identity refers to an individual’s knowledge of belonging and perceived emotional and value significance of group membership [42]. Social identity can act as the basis for both giving and receiving social support. Perceived social support can additionally promote the sense of shared identity and the subjective importance of one’s group membership [19,42,45,46]. Subsequently, social identity and group membership are associated with the internalization of group norms. The norms and attitudes shared within the group are internalized as personal standards and the individuals act accordingly [47]. On websites related to weight loss, nutrition, and exercise, users share body-appearance standards, which are demonstrated by the website content, and have discussions about ideal appearance and figure [18]. With these shared interests, the goals, the mutual interaction, and the social support that are exchanged among visitors, the websites have a social character. Thus, consistent with the Social Identity Theory approach, the perceived social support from the health-oriented websites can promote a sense of shared social identity and the perception of salience within the website group membership. Consequently, norms and standards regarding body appearance can be internalized even more.

### 1.4. Neuroticism

Neuroticism is defined in terms of the inclination to emotional reactivity, instability, perceived anxiety, and high vulnerability when coping with stress [33,48,49]. Individuals who are high in neuroticism are excitable, easily upset, and prone to experiences that are unpleasant [50]. They are also more sensitive to criticism; they experience higher levels of rejection; and they have lower self-esteem [51]. In prior research, neuroticism has been connected to the increased drive for thinness in women [52,53], to heightened food and body preoccupation [54], to body dissatisfaction [55], to the self-regulation of eating attitudes (e.g., food temptation) [56], and even to eating disorder diagnosis [48,57] and binge eating [58,59]. According to Fischer, Schreyer, Coughlin, Redgrave, and Guarda [52], the facets of neuroticism, including irritability and difficulty with emotional regulation, are risk factors for developing an ED. Moreover, disordered eating is associated with neuroticism because it can serve as a coping mechanism with which neurotic individuals deal with negative feelings [58,60].

In this study, we examine neuroticism as a risk factor for increased internalization, which can lead to a stronger drive for thinness. The link was proposed by Scoffier-Mériaux et al. [56], who hypothesized internalization as a mediator between neuroticism and unhealthy dieting behavior. This model was subsequently tested by Martin and Racine [49], who examined the mediating roles of thin and athletic-ideal internalization in association between neuroticism, body dissatisfaction, and compulsive exercise. Using the sample of 531 college students (58% women) aged 18–44, they found that thin-ideal internalization mediated the link between neuroticism and body dissatisfaction, and the internalization of athletic ideals mediated the effect of neuroticism on compulsive exercise. Moreover, several prior studies have found that neuroticism is associated with higher internalization [49,50,56,61]. To explain this link, Roberts and Good [50] suggest that women with increased neuroticism compare themselves to attractive people, and this comparison is more likely to result in negativity due to their emotional liability. This negative effect, which arises from the incongruity between the internalized body ideal and the actual body shape, can result in an increased drive for thinness, as has been proposed by previous studies [52,53]. Therefore, we hypothesize that internalization may be a mechanism through which neuroticism is positively linked to the drive for thinness in women.

### 1.5. Research Goals

This study focuses on the drive for thinness, which is considered a risk for women’s well-being. It aims to enhance our understanding of the risk factors that contribute to its development, specifically with regard to the influence of media and the role of individual factors in young women. Previous studies have shown that the media can have a negative effect on women because exposure to its content is associated with their desire to have a thin body shape [1,5,9]. However, these studies mainly investigated traditional media (i.e., TV, magazines) and pro-eating-disorder websites. There is a lack of research in health-oriented websites, which are currently popular. These websites display content that is associated with the drive for thinness, body dissatisfaction, and eating disturbances [4,5,23,24]. Therefore, our aim is to fill this gap and bring more insight into the association between visiting health-related websites and the drive for thinness among women. Furthermore, our study aims to enrich the Tripartite Influence Model [3], which is the theoretical framework that explains eating disturbances with socio-cultural factors, by incorporating neuroticism and perceived social support as individual factors. Specifically, we test whether web content internalization mediates the effect of these factors. We propose that increased neuroticism and perceived online social support positively affects web content internalization, which in turn affects the drive for thinness. Considering that disordered eating can be related to age and Body Mass Index [62,63,64], we also control for both of these factors.

## 2. Materials and Methods

### 2.1. Study Sample

This study uses data from a project which focused on the visitors of websites oriented toward nutrition, weight loss, and exercise. The data were collected through an online survey between May and October 2016. Participants were recruited with an invitation on 65 Czech websites, web magazines, social networking sites, blogs, and discussion forums that focused on weight loss, diet, eating habits, and exercise. The original sample comprised of 1002 respondents (81.6% women, aged 13 to 62, M = 24.8, SD = 6.9). The project was approved by the Research Ethics Committee of the University.

The current study focuses on a subsample of 445 young adult women, aged 18 to 29 (M = 23.5, SD = 3.1). Because the ideal of thinness is aimed mainly at women [1] and the drive for thinness and eating disorders are more prevalent in women [14], we focused on women in our study. Moreover, young adult women were the major part of the health-oriented website visitors in the project, and we did not have a sufficient amount of data from participants of other ages and genders. The original sample of women in the age range from 18 to 29 comprised of 632 participants. We excluded respondents based on their motivation for visiting health-oriented websites and because of missing data. We excluded women who reported that the reason for their website visits was because of the health issues of someone else (as indicated by the question *Do you visit the sites about nutrition or sports not for yourself, but mainly because you want to help with the nutrition or sport of another person (partner, child, parent, etc.)*?) (N = 37). In addition, participants with a substantial number of missing values for the key variables (N = 150) were excluded, and there were no significant age differences between our sample and excluded respondents (t = 0.37, *p* = 0.71)).

### 2.2. Measures

#### 2.2.1. Perceived Online Social Support

Perceived online social support was assessed using three items adapted from Graham, Papandonatos, Kang, Moreno, and Abrams [65]: *I get advice and support here that I would not get elsewhere*; *It is encouraging to know that there are other people making similar efforts (with regard to nutrition or sport)*; and *I feel that other visitors (or authors) of sites are giving me support*, with answers that ranged from 1 = Definitely does not apply to 4 = Definitely applies. A higher score indicated higher perceived support. The internal consistency was acceptable (ꙍ = 0.72, M = 2.8, SD = 0.7).

#### 2.2.2. Neuroticism

We measured neuroticism with three items from the short 15-item Big Five Inventory [66]. The items were *I worry a lot*; *I get nervous easily*; and *I remain calm in tense situations* (reverse scored). Participants answered on a six-point scale that ranged from 1 = Does not apply to 6 = Definitely applies. A higher score indicated higher neuroticism. The internal consistency was acceptable (ꙍ = 0.67, M = 3.7, SD = 1.1).

#### 2.2.3. Web Content Internalization

Internalization was measured using the question “To what extent do the following statements apply to you in regards to these sites?” with three items that were adapted from Cusumano and Thompson [67]: *I compare my appearance with people on these sites*; *I try to look like the people on these sites*; and *The content on these sites inspires me in how to look attractive*. Participants answered on a six-point scale that ranged from 1 = Does not apply to 6 = Definitely applies. A higher score indicated higher web content internalization. The internal consistency was satisfactory (ꙍ = 0.81, M = 2.4, SD = 0.8).

#### 2.2.4. Drive for Thinness

The Drive for Thinness subscale from Eating Disorder Inventory-3 [68] was used. The scale consisted of seven items (e.g., *I feel extremely guilty after overeating*; *I am preoccupied with the desire to be thinner*). Participants responded on a six-point scale that ranged from 1 = Never to 6 = Always. A higher score indicated a higher drive for thinness. The internal consistency was satisfactory (ꙍ = 0.86, M = 3.4, SD = 1.2). The latent variable was constructed with the parceling approach; specifically, we made three parcels, combining low-loading and high-loading items [69]. Parcels were computed as a mean of the items.

#### 2.2.5. BMI

Participants provided information about their current weight (in kilograms) and height (in centimeters). BMI was computed as follows: Weight (kg)/Height (m)^2^.

## 3. Results

We examined the correlations among the variables (Table 1): perceived online social support, neuroticism, web content internalization, and the drive for thinness. The results were as expected: the drive for thinness was positively correlated with online social support (*r* = 0.11, *p* = 0.03), web content internalization (*r* = 0.51, *p* < 0.001), and neuroticism (*r* = 0.23, *p* < 0.001). Web content internalization was positively associated with online social support (*r* = 0.24, *p* < 0.001) and neuroticism (*r* = 0.16, *p* < 0.001). Additionally, the drive for thinness was positively associated with BMI (*r* = 0.20, *p* < 0.001), but not with age (*r* = 0.02, *p* = 0.67).

**Figure 1:**
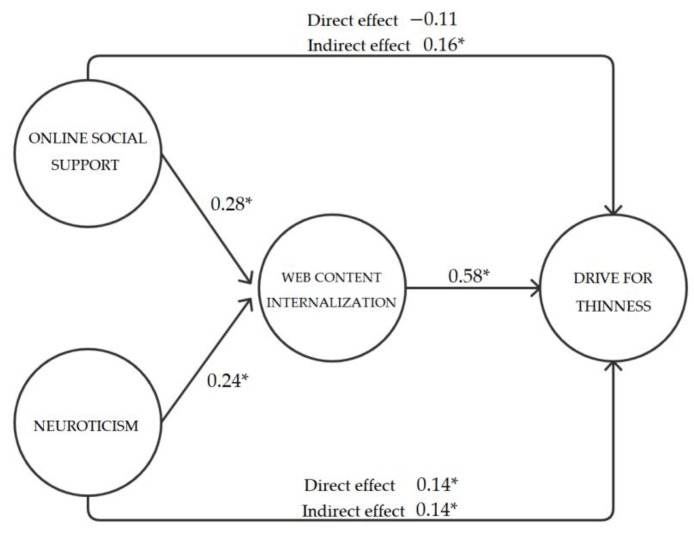
Path diagram with standardized regression coefficients (β). Note: * designates *p* < 0.05.

To test our presumptions, Structural Equation Modeling (SEM) was used with a Robust Maximum Likelihood (MLR) estimator. We used R software, and lavaan, semTools, and semPlot packages. We tested a model with indirect effects, predicting drive for thinness. We included neuroticism and online social support as predictors, the web content internalization as a mediator of the effect of neuroticism and social support, and age and BMI as controls. The model had an acceptable fit, CFI = 0.98, TLI = 0.97, RMSEA = 0.04. Results are displayed in Figure 1 and Table 2.

Perceived online social support from health-oriented websites predicted web content internalization (β = 0.28, *p* < 0.001). Perceived online social support did not have a strong direct effect on the drive for thinness, though the effect was weak and marginally significant (β = −0.11, *p* = 0.06; CI = −0.61; 0.01). Moreover, we found a significant indirect effect for online social support on the drive for thinness via web content internalization (β = 0.16, *p* = 0.001).

Neuroticism predicted web content internalization (β = 0.24, *p* < 0.001), and it had a direct effect on the drive for thinness (β = 0.14, *p* = 0.01). Moreover, we found a significant indirect effect for neuroticism on the drive for thinness through web content internalization (β = 0.14, *p* < 0.001). Therefore, the link between neuroticism and the drive for thinness was partially mediated by the web content internalization. Regarding controls, BMI positively predicted the drive for thinness (β = 0.17, *p* = 0.001), but there was no association between age and the drive for thinness (β = 0.02, *p* = 0.60).

## 4. Discussion

In our study, we examined the factors associated with the drive for thinness in young adult women who visited websites oriented toward weight loss, nutrition, and exercise. Specifically, we investigated the perceived online social support of other website visitors, the neuroticism, and the web content internalization of the body appearance standards, and their direct and indirect effects on the drive for thinness. Our objective was to investigate whether the web content internalization mediates the links among the perceived online social support, the neuroticism, and the drive for thinness. We found support for our presumption: both online support and neuroticism were positively linked with the tendency for internalization, which, in turn, increased the drive for thinness.

In our data, we found a substantial connection between internalization and the drive for thinness. Our findings are in line with the Tripartite Influence Model [3,31,32,33], which suggests that body image concerns and eating disorders are affected by socio-cultural factors (e.g., media pressure, parental criticism, peer criticism) and indirectly through the internalization of the medialized body ideals. Moreover, we enriched the propositions of the Tripartite Influence Model [3] by including individual factors. This line of research was recently developed in studies that focused on perfectionism, self-esteem, depression, and anxiety [30,31,32,33,34,70]. This focus helps to better understand the risk factors, which strengthen the tendency for internalization.

Specifically, we found that perceived support increased the drive for thinness via its reinforcement of internalization. Our findings correspond to knowledge regarding ED (eating disorders) online groups, in which perceived support was connected to a higher sense of belonging and the acceptance of thin-ideal norms [20,22,39,71]. ED online groups and communities act as an important source of support that can be difficult to obtain elsewhere for individuals who struggle with ED and body image concerns [39,41]. However, support received from these online groups can be detrimental to women’s health because it endorses negative attitudes toward their bodies and promotes extremely thin body shapes as attainable standards. Haas et al. [72] examined the social support on pro-anorexia websites and discovered that visitors received support for eating restraint and reinforcement for their negative views of themselves and their bodies. Sowles et al. [73] pointed out that members of the pro-ED online community disseminate images that depict thin body shapes and promote the thin ideal by labeling them as their desired goals. Similar findings emerged from a study by Marcus [40], who found that members of a pro-anorexic community shared photos of extremely thin bodies to motivate users to maintain their diets and to outline the beauty standards of the group. In this manner, women are encouraged to adopt body appearance standards that lead to a desire for a thin body. The findings of our study suggest that these processes apply not only to ED online groups, but to health-related websites as well.

Health-oriented websites, with their opportunities for social interaction (e.g., discussion with other users about specific health-related topics, personal messages, inspiration, sharing experiences, memories, feelings), enable visitors to receive social support. The perceived social support is associated with the acceptance of group norms due to the higher subjective salience of the social group to which the individuals belong [40,44,47]. In line with social identity theory [44], the stronger identification with a group would result in the acceptance of group norms and, in the case of websites focusing on nutrition and fitness—these probably supported the thin and fitness-oriented images of the ideal body. Thus, though the perceived support is often seen as a positive aspect of online interaction, in these instances, it may result in negative outcomes. However, when interpreting these results, the limitations of this study should be taken into consideration. Due to the correlational nature of the data used, it was not possible to draw causal conclusions. Thus, the association between online social support and the drive for thinness may work in the opposite direction, meaning that women with a stronger drive for thinness may more often seek social support for their goals and efforts in the online space and, specifically, via health-oriented websites.

Moreover, this finding should also be compared to the results for the direct effect of support on the drive for thinness. This effect was rather weak and just marginally significant; however, it may indicate that the role of support is diverse. If we disentangle the indirect effect that positively affects the drive for thinness from the direct effect, we find that support negatively affected the drive for thinness. To interpret this finding, we should acknowledge that perceived support helps to increase overall well-being [74,75,76], which decreases the tendency for unhealthy and disordered eating habits [77,78]. Thus, perceived online social support can actually function as both a risk and a protective factor. On one hand, it may contribute to the development of the drive for thinness via increased internalization. On the other hand, it may also serve as a buffer for this negative effect, probably via the increase of overall well-being, which was not included in this study. This presumption could be pursued in future examinations.

Thus, we still need to consider other factors which underlie the internalization of the web content. Our study focused on neuroticism, which showed to be positively linked to the drive for thinness and also had an indirect effect via internalization. Therefore, the effect of neuroticism on women’s drive for thinness was partially mediated by the internalization of the body appearance standards displayed on health-oriented websites. In line with prior studies [49,50,52,53,56,61], our findings showed that people with heightened neuroticism are more prone to accepting the norms, and, probably because of the increased tendency for social comparison, tend more to strive to be thin.

However, besides the mediated effect, we also found a direct positive link to the drive for thinness. This suggests that increased internalization is not the only mechanism through which people with neurotic traits can be more at risk. However, considering that we found support for the tendency for heightened internalization from the websites, and upon the propositions of the Tripartite Influence Model [3], we could expect that the mechanism could be similar in relation to parental and peer norms, which have not been measured in this study. This poses one of the limitations for our study.

Concerning other limitations, it should be stressed that we used cross-sectional correlational data based on a sample that was self-selected through health-oriented websites. Thus, though we examined the proposed model for the mechanisms to increase the drive for thinness, the research design complicates drawing causal conclusions. Future research should implement a longitudinal research design to make more reliable causal conclusions and to capture potential reciprocal associations. Moreover, we were not able to control for the effects of additional variables on the drive for thinness. These are factors (e.g., body dissatisfaction) [79] that are related to the drive for thinness and disordered eating, and it would be appropriate to control for their effects to obtain more accurate results. Furthermore, we do not have information about the specific content that respondents encountered. It would be useful to incorporate objective measures and directly observe the effects of participants’ exposure to online content. Finally, although the thin ideal displayed in the media and the related drive for thinness is more prominent in women [1], future research could focus on men, their internalization of the body appearance norms, and their motivations for body change.

In the current study, our aim was to propound a model that comprises of the individual factors that affect women’s drive for thinness. Based on our findings, we can formulate several implications. According to the theory and the available data, we propose the following processes: online social support from the visitors of health-oriented websites and neuroticism affect the drive for thinness, and these links are mediated by the internalization of body appearance standards. Thus, alongside previous research in this area [1,8,15,35,36], our study supported the predictive role of internalization in the drive for thinness among women. Specifically, our study provided insight into the internalization of the content of health-oriented websites, which had not been sufficiently investigated and had not been taken into account in relation to women’s drive for thinness. Our results imply that it is crucial to acknowledge health-oriented websites and their potential impact on women, especially in the context of the internalization of body appearance norms. Health-oriented websites, which are not generally acknowledged as harmful to women’s body image, can be a significant source of body appearance norms and subsequent body image concerns [18]. As was discovered in the current study, women internalize body ideals from health-oriented websites and this, in turn, increases their drive for thinness. This connection should be actively acknowledged by health-care professionals. It is important for professionals to ask their clients who have ED-related problems about their technology usage and to provide them with space to talk about it [80]. Thus, in the context of the current study, health-care professional should discuss with clients who are struggling with EDs their usage of health-oriented websites, specifically with a focus on their exposure to the thin- or fitness-ideal content.

We also showed that both online support and neuroticism present risk factors because they can increase the tendency for internalization and, in turn, increase the drive for thinness. Therefore, it is important to be aware of the possible negative effect that online social support may have on women and to address it when preventing or reducing the drive for thinness. However, the findings of this study showed that online social support can function both as a risk and a protective factor. Thus, when discussing the use of health-oriented websites with ED clients, it is important to disentangle the different forms of social support that women receive from the visitors of these platforms. In addition, neurotic individuals experience higher levels of negative emotions and stress, which makes them more susceptible [52,53]. Based on our results, we suggest that preventive health programs, intervention, individual psychotherapy, counseling, and other health policies can be focused on the reduction of the negative emotions and stress in women. These can also help the reduction of the internalization of the body appearance standards promoted on health-oriented websites.

## 5. Conclusions

This study focused on the factors associated with the drive for thinness in young adult women who visited weight loss, nutrition, and exercise websites. These platforms are currently of high use, yet they have not been sufficiently studied in relation to eating disturbances. We examined the direct and indirect effects of perceived online social support from fellow website visitors, neuroticism, and the web content internalization of the body appearance standards on the drive for thinness. Our findings supported the predictive role of web content internalization on the drive for thinness in women. Moreover, we showed that the perceived online support from the health-oriented websites and neuroticism can pose risk factors because they are associated with a higher tendency for internalization and, in turn, with a stronger drive for thinness. Our results indicate that it is crucial to acknowledge health-oriented websites and their potential impact on women and their drive for thinness, especially in the context of the internalization of body appearance standards. We also discuss the role of social support, and its double role of risk and protection. Our findings can be used to establish prevention and intervention efforts to help individuals who struggle with body image and eating disturbances.

## Figures and Tables

**Table 1:** Correlations among variables.

Examined Variables	Drive for Thinness	Online Support	Neuroticism	Internalization	BMI	Age
Drive for thinness						
Online support	0.11 *					
Neuroticism	0.23 *	−0.01				
Internalization	0.51 *	0.24 *	0.16 *			
BMI	0.20 *	0.07	0.10 *	0.02		
Age	0.02	0.002	−0.04	−0.05	0.15 *	

Note: * designates *p* < 0.05.

**Table 2:** Structural Equation Modeling (SEM) predicting the drive for thinness and web content internalization.

Drive for Thinness		b	SE	*p*	β	CI
BMI		0.05	0.01	0.001	0.17	(0.03; 0.07)
Age		0.008	0.02	0.60	0.02	(−0.03; 0.05)
Web content internalization		0.77	0.09	<0.001	0.58	(0.59; 0.95)
Online social support	Direct effect	−0.30	0.16	0.06	−0.11	(−0.61; 0.01)
	Indirect effect	0.44	0.13	0.001	0.16	(0.19; 0.69)
Neuroticism	Direct effect	0.14	0.06	0.01	0.14	(0.02; 0.26)
	Indirect effect	0.14	0.04	<0.001	0.14	(0.06; 0.22)
**Web content internalization**		**b**	**SE**	***p***	**β**	**CI**
Online social support		0.57	0.16	<0.001	0.28	(0.26; 0.88)
Neuroticism		0.18	0.05	<0.001	0.24	(0.08; 0.28)

## References

[1] Pritchard M., Cramblitt B. (2014). Media Influence on Drive for Thinness and Drive for Muscularity. Sex Roles.

[2] Warren C.S., Gleaves D.H., Cepeda-Benito A., Fernandez M.D.C., Rodriguez-Ruiz S. (2005). Ethnicity as a Protective Factor against Internalization of a Thin Ideal and Body Dissatisfaction. Int. J. Eat. Disord..

[3] Thompson J.K., Heinberg L.J., Altabe M., Tantleff-Dunn S. (1999). Exacting Beauty: Theory, Assessment, and Treatment of Body Image Disturbance.

[4] Harper K., Sperry S., Thompson J.K. (2008). Viewership of Pro-Eating Disorder Websites: Association with Body Image and Eating Disturbances. Int. J. Eat. Disord..

[5] Juarez L., Soto E., Pritchard M.E. (2012). Drive for Muscularity and Drive for Thinness: The Impact of Pro-Anorexia Websites. Eat. Disord..

[6] Jones M.E., Blodgett Salafia E.H., Hill B.D. (2019). The Effect of Parental Warmth on Girls’ Drive for Thinness: Do Both Parents Matter?. J. Child Fam. Stud..

[7] De Pasquale C., Pistorio M.L., Tornatore E., De Berardis D., Fornaro M. (2013). The Relationship between Drive to Thinness, Conscientiousness and Bulimic Traits during Adolescence: A Comparison between Younger and Older Cases in 608 Healthy Volunteers. Ann. Gen. Psychiatry.

[8] Fernandez S., Pritchard M. (2012). Relationships between Self-Esteem, Media Influence and Drive for Thinness. Eat. Behav..

[9] Tiggemann M. (2006). The Role of Media Exposure in Adolescent Girls’ Body Dissatisfaction and Drive for Thinness: Prospective Results. J. Soc. Clin. Psychol..

[10] Sands R. (2000). Reconceptualization of Body Image and Drive for Thinness. Int. J. Eat. Disord..

[11] Wiederman M.W., Pryor T.L. (2000). Body Dissatisfaction, Bulimia, and Depression among Women: The Mediating Role of Drive for Thinness. Int. J. Eat. Disord..

[12] Brunet J., Sabiston C.M., Dorsch K.D., McCreary D.R. (2010). Exploring a Model Linking Social Physique Anxiety, Drive for Muscularity, Drive for Thinness and Self-Esteem among Adolescent Boys and Girls. Body Image.

[13] Warren C.S., Holland S., Billings H., Parker A. (2012). The Relationships between Fat Talk, Body Dissatisfaction, and Drive for Thinness: Perceived Stress as a Moderator. Body Image.

[14] Galmiche M., Déchelotte P., Lambert G., Tavolacci M.P. (2019). Prevalence of Eating Disorders over the 2000–2018 Period: A Systematic Literature Review. Am. J. Clin. Nutr..

[15] Tiggemann M., Miller J. (2010). The Internet and Adolescent Girls’ Weight Satisfaction and Drive for Thinness. Sex Roles.

[16] Bissonnette-Maheux V., Provencher V., Lapointe A., Dugrenier M., Dumas A.A., Pluye P., Straus S., Gagnon M.P., Desroches S. (2015). Exploring Women’s Beliefs and Perceptions About Healthy Eating Blogs: A Qualitative Study. J. Med. Internet Res..

[17] Ransom D.C., La Guardia J.G., Woody E.Z., Boyd J.L. (2010). Interpersonal Interactions on Online Forums Addressing Eating Concerns. Int. J. Eat. Disord..

[18] Boepple L., Thompson J.K. (2014). A Content Analysis of Healthy Living Blogs: Evidence of Content Thematically Consistent with Dysfunctional Eating Attitudes and Behaviors. Int. J. Eat. Disord..

[19] Smahel D., Machackova H., Smahelova M., Cevelicek M., Almenara C.A., Holubcikova J. (2018). Digital Technology, Eating Behaviors, and Eating Disorders.

[20] Flynn M.A., Stana A. (2012). Social Support in a Men’s Online Eating Disorder Forum. Int. J. Mens. Health.

[21] Hwang K.O., Ottenbacher A.J., Green A.P., Cannon-Diehl M.R., Richardson O., Bernstam E.V., Thomas E.J. (2010). Social Support in an Internet Weight Loss Community. Int. J. Med. Inform..

[22] McCormack A. (2010). Individuals with Eating Disorders and the Use of Online Support Groups as a Form of Social Support. CIN Comput. Inform. Nurs..

[23] Custers K., Van den Bulck J. (2009). Viewership of Pro-Anorexia Websites in Seventh, Ninth and Eleventh Graders. Eur. Eat. Disord. Rev..

[24] Rouleau C.R., Von Ranson K.M. (2011). Potential Risks of Pro-Eating Disorder Websites. Clin. Psychol. Rev..

[25] Turja T., Oksanen A., Kaakinen M., Sirola A., Kaltiala-Heino R., Räsänen P. (2017). Proeating Disorder Websites and Subjective Well-Being: A Four-Country Study on Young People. Int. J. Eat. Disord..

[26] Ging D., Garvey S. (2018). ‘Written in These Scars Are the Stories I Can’t Explain’: A Content Analysis of pro-Ana and Thinspiration Image Sharing on Instagram. New Media Soc..

[27] Carrotte E.R., Vella A.M., Lim M.S. (2015). Predictors of “Liking” Three Types of Health and Fitness-Related Content on Social Media: A Cross-Sectional Study. J. Med. Internet Res..

[28] Eurostat Women Aged 16–29 Using the Internet For Seeking Health-Related Information. https://appsso.eurostat.ec.europa.eu/nui/show.do?dataset=isoc_ci_ac_i&lang=en.

[29] Eurostat Women Aged 16–29 Using the Internet in Last Three Months. https://appsso.eurostat.ec.europa.eu/nui/show.do?dataset=isoc_ci_ifp_iu&lang=en.

[30] Van Den Berg P., Thompson J.K., Obremski-Brandon K., Coovert M. (2002). The Tripartite Influence Model of Body Image and Eating Disturbance A Covariance Structure Modeling Investigation Testing the Mediational Role of Appearance Comparison. J. Psychosom. Res..

[31] Huxley C.J., Halliwell E., Clarke V. (2015). An Examination of the Tripartite Influence Model of Body Image: Does Women’s Sexual Identity Make a Difference?. Psychol. Women Q..

[32] Keery H., van den Berg P., Thompson J.K. (2004). An Evaluation of the Tripartite Influence Model of Body Dissatisfaction and Eating Disturbance with Adolescent Girls. Body Image.

[33] Pokrajac-bulian A. (2008). Thin-Ideal Internalization and Comparison Process as Mediators of Social Influence and Psychological Functioning in the Development of Disturbed Eating Habits in Croatian College Females. Psychol. Top..

[34] Jones D.C., Vigfusdottir T.H., Lee Y. (2004). Body Image and the Appearance Culture among Adolescent Girls and Boys: An Examination of Friend Conversations, Peer Criticism, Appearance Magazines, and the Internalization of Appearance Ideals. J. Adolesc. Res..

[35] Gilbert S.C., Crump S., Madhere S., Schutz W. (2009). Internalization of the Thin Ideal as a Predictor of Body Dissatisfaction and Disordered Eating in African, African-American, and Afro-Caribbean Female College Students. J. Coll. Stud. Psychother..

[36] Low K.G., Charanasomboon S., Brown C., Hiltunen G., Long K., Reinhalter K., Jones H. (2003). Internalization of the Thin Ideal, Weight and Body Image Concerns. Soc. Behav. Pers..

[37] Peebles R., Wilson J.L., Litt I.F., Hardy K.K., Lock J.D., Mann J.R., Borzekowski D.L.G. (2012). Disordered Eating in a Digital Age: Eating Behaviors, Health, and Quality of Life in Users of Websites with pro-Eating Disorder Content. J. Med. Internet Res..

[38] Reijo S. (2010). Dietary Blogs as Sites of Informational and Emotional Support. Inf. Res..

[39] Tong S.T., Heinemann-LaFave D., Jeon J., Kolodziej-Smith R., Warshay N. (2013). The Use of Pro-Ana Blogs for Online Social Support. Eat. Disord..

[40] Marcus S.R. (2016). Thinspiration vs. Thicksperation: Comparing pro-Anorexic and Fat Acceptance Image Posts on a Photo-Sharing Site. Cyberpsychology.

[41] Kendal S., Kirk S., Elvey R., Catchpole R., Pryjmachuk S. (2017). How a Moderated Online Discussion Forum Facilitates Support for Young People with Eating Disorders. Health Expect..

[42] Chiu C.M., Huang H.Y., Cheng H.L., Sun P.C. (2015). Understanding Online Community Citizenship Behaviors through Social Support and Social Identity. Int. J. Inf. Manag..

[43] Tajfel H., Turner J.C. (1979). An integrative theory of intergroup conflict. The Social Psychology of Intergroup Relation.

[44] Tajfel H. (2010). Social Identity and Intergroup Relations.

[45] Deaux K. (1993). Reconstructing Social Identity. Soc. Personal. Soc. Psychol..

[46] Haslam S.A., O’Brien A., Jetten J., Vormedal K., Penna S. (2005). Taking the strain: Social identity, social support, and the experience of stress. Br. J. Soc. Psychol..

[47] Stets J.E., Burke P.J. (2000). Identity Theory and Social Identity Theory. Soc. Psychol. Q..

[48] Gual P., Pérez-Gaspar M., Martinez-Gonzalez M.A., Lahortiga F., de Irala-Estévez J., Cervera-Enguix S. (2002). Self-Esteem, Personality, and Eating Disorders: Baseline Assessment of a Prospective Population-Based Cohort. Int. J. Eat. Disord..

[49] Martin S.J., Racine S.E. (2017). Personality Traits and Appearance-Ideal Internalization: Differential Associations with Body Dissatisfaction and Compulsive Exercise. Eat. Behav..

[50] Roberts A., Good E. (2010). Media Images and Female Body Dissatisfaction: The Moderating Effects of the Five-Factor Traits. Eat. Behav..

[51] Rozgonjuk D., Ryan T., Kuljus J.K., Täht K., Scott G.G. (2019). Social Comparison Orientation Mediates the Relationship between Neuroticism and Passive Facebook Use. Cyberpsychology.

[52] Fischer L.K., Schreyer C.C., Coughlin J.W., Redgrave G.W., Guarda A.S. (2017). Neuroticism and Clinical Course of Weight Restoration in a Meal-Based, Rapid-Weight Gain, Inpatient-Partial Hospitalization Program for Eating Disorders. Eat. Disord..

[53] Kjelsås E., Augestad L.B. (2004). Gender, Eating Behavior, and Personality Characteristics in Physically Active Students. Scand. J. Med. Sci. Sports.

[54] Ellickson-Larew S., Naragon-Gainey K., Watson D. (2013). Pathological Eating Behaviors, BMI, and Facet-Level Traits: The Roles of Conscientiousness, Neuroticism, and Impulsivity. Eat. Behav..

[55] MacNeill L.P., Best L.A., Davis L.L. (2017). The Role of Personality in Body Image Dissatisfaction and Disordered Eating: Discrepancies between Men and Women. J. Eat. Disord..

[56] Scoffier-Mériaux S., Falzon C., Lewton-Brain P., Filaire E., d’arripe-Longueville F. (2015). Big Five Personality Traits and Eating Attitudes in Intensively Training Dancers: The Mediating Role of Internalized Thinness Norms. J. Sports Sci. Med..

[57] Cervera S., Lahortiga F., Martinez-Gonzalez M.A., Gual P., de Irala-Estevez J., Alonso Y. (2003). Neuroticism and Low Self-Esteem as Risk Factors for Incident Eating Disorders in a Prospective Cohort Study. Int. J. Eat. Disord..

[58] Izydorczyk B. (2012). Neuroticism and Compulsive Overeating (A Comparative Analysis of the Level of Neuroticism and Anxiety in a Group of Females Suffering from Psychogenic Binge Eating, and in Individuals Exhibiting No Mental or Eating Disorders). Arch. Psychiatry Psychother..

[59] Koren R., Munn-Chernoff M.A., Duncan A.E., Bucholz K.K., Madden P.A.F., Heath A.C., Agrawal A. (2014). Is the Relationship between Binge Eating Episodes and Personality Attributable to Genetic Factors?. Twin Res. Hum. Genet..

[60] Henderson Z.B., Fox J.R.E., Trayner P., Wittkowski A. (2019). Emotional Development in Eating Disorders: A Qualitative Metasynthesis. Clin. Psychol. Psychother..

[61] Heinberg L.J., Coughlin J.W., Pinto A.M., Haug N., Brode C., Guarda A.S. (2008). Validation and Predictive Utility of the Sociocultural Attitudes toward Appearance Questionnaire for Eating Disorders (SATAQ-ED): Internalization of Sociocultural Ideals Predicts Weight Gain. Body Image.

[62] Chaudhari B., Tewari A., Vanka J., Kumar S., Saldanha D. (2017). The Relationship of Eating Disorders Risk with Body Mass Index, Body Image and Self-Esteem among Medical Students. Ann. Med Health Sci. Res..

[63] Dada G., Feixas G., Compañ V., Montesano A. (2012). Self-Construction, Cognitive Conflicts, and Disordered Eating Attitudes in Young Women. J. Constr. Psychol..

[64] Rojo-Moreno L., Rubio T., Plumed J., Barberá M., Serrano M., Gimeno N., Conesa L., Ruiz E., Rojo-Bofill L., Beato L. (2013). Teasing and Disordered Eating Behaviors in Spanish Adolescents. Eat. Disord..

[65] Graham A.L., Papandonatos G.D., Kang H., Moreno J.L., Abrams D.B. (2011). Development and validation of the online social support for smokers scale. J. Med Internet Res..

[66] Lang F.R., John D., Lüdtke O., Schupp J., Wagner G.G. (2011). Short assessment of the Big Five: Robust across survey methods except telephone interviewing. Behav. Res. Methods.

[67] Cusumano D.L., Thompson J.K. (2001). Media influence and body image in 8–11-year-old boys and girls: A preliminary report on the multidimensional media influence scale. Int. J. Eat. Disord..

[68] Garner D.M. (2004). Eating Disorder Inventory-3. Professional Manual.

[69] Little T.D., Cunningham W.A., Shahar G., Widaman K.F. (2002). To Parcel or Not to Parcel: Exploring the Question, Weighing the Merits. Struct. Equ. Model..

[70] Menzel J.E., Sperry S.L., Small B., Thompson J.K., Sarwer D.B., Cash T.F. (2011). Internalization of Appearance Ideals and Cosmetic Surgery Attitudes: A Test of the Tripartite Influence Model of Body Image. Sex Roles.

[71] McKinley C.J. (2009). Investigating the influence of threat appraisals and social support on healthy eating behavior and drive for thinness. Health Commun..

[72] Haas S.M., Irr M.E., Jennings N.A., Wagner L.M. (2011). Communicating Thin: A Grounded Model of Online Negative Enabling Support Groups in the pro-Anorexia Movement. New Media Soc..

[73] Sowles S.J., McLeary M., Optican A., Cahn E., Krauss M.J., Fitzsimmons-Craft E.E., Wilfley D.E., Cavazos-Rehg P.A. (2018). A Content Analysis of an Online Pro-Eating Disorder Community on Reddit. Body Image.

[74] Espeleta H.C., Beasley L., Bohora S., Ridings L.E., Silovsky J.F. (2019). Depression in Latina mothers: Examining the roles of acculturation, enculturation, social support, and family resources. Cult. Divers. Ethn. Minority Psychol..

[75] Grieve R., Indian M., Witteveen K., Anne Tolan G., Marrington J. (2013). Face-to-face or Facebook: Can social connectedness be derived online?. Comput. Hum. Behav..

[76] Ibrahim N., Che Din N., Ahmad M., Amit N., Ghazali S.E., Wahab S., Abdul Kadir N., Halim F.W., Halim M.R.T.A. (2019). The role of social support and spiritual wellbeing in predicting suicidal ideation among marginalized adolescents in Malaysia. BMC Public Health.

[77] Fitzsimmons E.E., Bardone-Cone A.M. (2011). Coping and social support as potential moderators of the relation between anxiety and eating disorder symptomatology. Eat. Behav..

[78] Wonderlich-Tierney A.L., Vander Wal J.S. (2010). The effects of social support and coping on the relationship between social anxiety and eating disorders. Eat. Behav..

[79] Keski-Rahkonen A., Bulik C.M., Neale B.M., Rose R.J., Rissanen A., Kaprio J. (2005). Body Dissatisfaction and Drive for Thinness in Young Adult Twins. Int. J. Eat. Disord..

[80] Šmahelová M., Čevelíček M., Nehybková E., Šmahel D., Čermák I. (2019). Is It Important to Talk about Technologies with Eating Disorder Clients? The Health-Care Professional Perspective. Health Commun..

